# Cytokine Patterns in Brain Tumour Progression

**DOI:** 10.1155/2013/979748

**Published:** 2013-06-24

**Authors:** Radu Albulescu, Elena Codrici, Ionela Daniela Popescu, Simona Mihai, Laura Georgiana Necula, Daniel Petrescu, Mihaela Teodoru, Cristiana Pistol Tanase

**Affiliations:** ^1^Victor Babes National Institute of Pathology, 99-101 Splaiul Independentei, 050096 Bucharest, Romania; ^2^National Institute for Chemical Pharmaceutical R&D, 112 Calea Vitan, 031299 Bucharest, Romania; ^3^Stefan S Nicolau Institute of Virology, 285 Soseaua Mihai Bravu, 030304 Bucharest, Romania; ^4^Neurology and Neurovascular Diseases National Institute, 10-12 Soseaua Berceni, 041914 Bucharest, Romania; ^5^Elias Emergency University Hospital, 19 Bulevardul Marasti, 011462 Bucharest, Romania

## Abstract

Inflammation represents the immune system response to external or internal aggressors such as injury or infection in certain tissues. The body's response to cancer has many parallels with inflammation and repair; the inflammatory cells and cytokines present in tumours are more likely to contribute to tumour growth, progression, and immunosuppression, rather than in building an effective antitumour defence. Using new proteomic technology, we have investigated serum profile of pro- (IL-1**β**, IL-6, IL-8, IL-12, GM-CSF, and TNF-**α**) and anti-inflammatory cytokines (IL-4, IL-10), along with angiogenic factors (VEGF, bFGF) in order to assess tumoural aggressiveness. Our results indicate significant dysregulation in serum levels of cytokines and angiogenic factors, with over threefold upregulation of IL-6, IL-1**β**, TNF-**α**, and IL-10 and up to twofold upregulation of VEGF, FGF-2, IL-8, IL-2, and GM-CSF. These molecules are involved in tumour progression and aggressiveness, and are also involved in a generation of disease associated pain.

## 1. Introduction

Glioblastomas are the most aggressive type of intracranial tumours, highly resistant to combined treatment, in patients displaying a median survival time of 15 months [1]. The molecular mechanisms underlying these clinical features are the existence of specific genetic and molecular profiles of these tumour cells. Recent reports show genomic instability (especially in tumours from short-term survival patients), chromosomal alterations, somatic mutations, and polymorphisms [2]. Knowing this particular brain tumour cell, one can wonder if, besides the intrinsic cellular features, the inflammatory milieu triggered by the development of such a tumour cannot influence the particular clinical development in glioblastomas as well.

The relationship between inflammation and cancer has first been suggested in modern time, by Virchow in 1863, who found “lymphoreticular infiltrates” in neoplastic tissues, consequently suggesting that these reflect the origin of cancer of sites of chronic inflammation. Massive experimental proofs appeared in the recent years to support Virchow's concept [3]. 

In a synthetic formulation, inflammation is defined as “the seventh hallmark of cancer”, by Colotta et al. [4]. The body's response to cancer has many analogies with inflammation and repair; the inflammatory cells and cytokines present in tumours are more likely to contribute to tumour growth, progression, and immunosuppression, rather than in building an effective antitumour defence. Cancer susceptibility and severity are often associated with functional polymorphisms in cytokine genes. As plastically described by Balkwill and Mantovani, if genetic damage is the “match that lights the fire” of cancer, some types of inflammation may provide the “fuel that feeds the flames” [3]. 

Tumour initiation and progression is a complex process involving genomic mutations, micro environmental factors, and inflammatory mediators. Within the tumour environment inflammatory markers are responsible for cell proliferation, tumour invasion, marked angiogenesis, and suppression of certain immune functions [5].

Inflammation represents the immune system response to external or internal aggressors, such as injury or infection in certain tissues. Typical signs of inflammation include swelling, redness, pain, temperature rise, and subsequently loss of function. Numerous studies have shown that the majority of tumour tissues are associated with inflammatory signs. However, a clear connection between inflammation and cancer has yet to be demonstrated.

Glioblastoma represents the most common and lethal primary brain tumour. The prognosis is poor, especially for higher grade glioma—the most common primary neoplasm of the central nervous system, composing over 40% of all such tumours, with an incidence ranging from 8% to 27% [6].

A broad array of cytokines displays modified expression in cancers, including glioblastoma multiforme [7, 8].

The changes arise from the interaction of tumour cells and nontumour cells, like macrophages, lymphocytes, or stromal cells, and provide regulatory support for tumour growth, angiogenesis, invasion, and metastasis [8–10].

The vascular system of brain cancers inappropriately expresses membrane proteins, resulting in blood extravasation. The production of inflammatory mediators (such as cytokines and nitric oxide), and tumour hypoxia have been involved in these effects [11].

**Figure 1:**
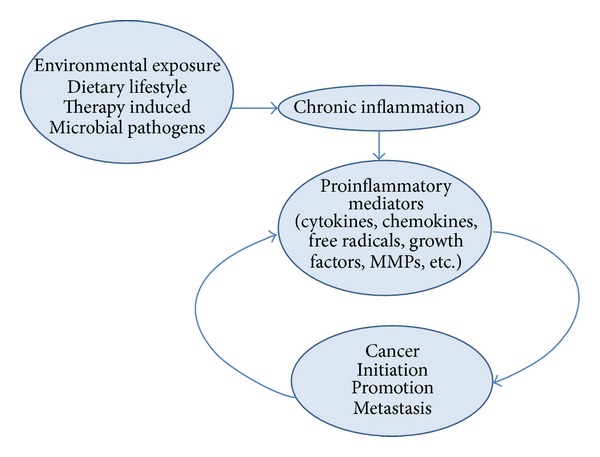
Implication of chronic inflammation in different stages of tumour development. Mediators of inflammation, triggered by different processes, may stimulate premalignant cell proliferation, angiogenesis, and metastasis. Reversely, tumour cells have the ability to stimulate other cells or to produce by themselves pro inflammatory and pro-angiogenic factors.

Pain belongs to the “classical” markers of inflammation, described over 2000 years ago by Aulus Celsus (*calor, rubor, tumour et dolor*). Various molecular actors of inflammation, including mediators of pain have been described in the recent years; a key role appears to be played by cytokines, which sometimes appear to conduct the orchestra of small molecule mediators, such as nitric oxide and prostaglandins. Many studies show that inflammation may be involved in different stages of tumour development. Several cancer risk factors like cigarette smoke, alcohol and growth factors can activate signaling pathways related with inflammation (such as NF*κ*B and STAT3 signaling). Some chronic infections lead to inflammatory conditions and are associated with carcinogenesis (e.g., hepatitis B virus). Chemotherapeutic agents and gamma irradiation can also interfere in the regulation of expression of some genes implicated in inflammation, survival, proliferation, invasion, angiogenesis, and cancer metastasis [12]. Inflammation may be involved in carcinogenesis through mutations, genomic instability, and epigenetic modifications [13]. Also, inflammation can participate in premalignant cells proliferation, stimulate angiogenesis, and promote metastatic spread (Figure 1). 

 Among the cytokines, often found to be over expressed at tumour level, IL1-beta, TNF-alpha, IL-6, IL-10, IFN-gamma, CX3CL1, to name just a few, have been closely related to pain for a long time [14–16].

In the case of glioblastoma, headache is one of the most frequently claimed signs by the patients, but also, very often the diagnostic is set too late for a successful therapeutical approach. 

Acknowledging the worldwide research effort in the field of glioblastoma, we have embarked in the study of circulatory cytokines to pinpoint the serum inflammatory pattern that can characterize the glioblastoma patient's evolution. Therefore, our study investigated the serum levels of several pro- and anti-inflammatory cytokines and of angiogenic factors in brain tumour patients diagnosed in stages III and IV (glioblastoma), in order to establish their roles and behaviour in tumour progression.

## 2. Material and Method

### 2.1. Patients and Samples

Samples (serum) were collected from 55 patients with glioblastoma (28 men and 27 women; mean ages: 58 and 62 years, resp., range: 37–79 years) from Neurology and Neurovascular Diseases National Institute, Elias Hospital Neurosurgery Department and 20 controls (healthy individuals with no known history of inflammatory or neoplastic diseases, 12 men and 8 women; mean age: 57 years, range: 25–70 years). Written informed consent has been obtained upon sample prelevation according to Helsinki II Declaration and Ethics Committee of Victor Babes National Institute of Pathology that has approved the study. The collection of total peripheral blood from patients and controls has been achieved in vacutainers (Systems, Becton Dickinson) without anticoagulant. Serum was aliquoted and stored at −80°C until analysis.

### 2.2. xMAP Analysis and ELISA

The xMAP assay was performed according to the manufacturers' protocols, and the plates were analysed using Luminex 200 system. Cytokines levels and angiogenic factors were determined using the Human cytokine 12-plex Kit, with 12 analyte-specific bead sets (simultaneous quantification)—pro-inflammatory IL-1*β*, IL-2, IL-6, IL-8, TNF*α*, GM-CSF, and INF*γ*, anti-inflammatory IL-4, IL-10, and IL-12, and angiogenic factors VEGF and FGF-2. Multiplex data acquisition and analysis were performed using STarStation 2.3. Triplicate samples were used for all specimens. Values for individual proteins measured by this multiplexed protein array technology have been shown to correlate with single ELISA measurements. 

Immunoenzymatic ELISA analysis was performed with Quantikine (R&D Systems). Serum level of growth factors was determined according to the manufacturer's protocol. All samples were assayed in triplicate, and the mean values of cytokines were taken into account. Optical density was measured at 450 nm on an Anthos Zenith 3100 multimode micro plate reader. Minimum detectable concentrations were found to be less than 9.0 pg/mL for VEGF and less than 3.0 pg/mL for bFGF.

### 2.3. Statistical Analysis

Data were collected and expressed as the mean ± standard error of three independent repeats. Differences between groups were analysed by One Way Anova; *P* values less than 0.05 were considered statistically significant; Pearson correlation (r, p) was used to explore the association between cytokine expressions. Statistical analysis was performed using SPSS 19.0 software.

## 3. Results and Discussion

From multiplex assay (Luminex 200) a strong overexpression was detected for IL-6, IL-1*β*, TNF-*α*, and IL-10 (over 3-fold stimulation in glioblastoma patients). Significant up-regulation (up to 2-fold) was found for VEGF, FGF-2, IL-8, IL-2, and GM-CSF. Cytokines expression was significantly higher and strongly correlated with tumour grade, proliferation markers, and clinical aggressiveness in glioblastomas. Comparing the patient groups and control for growth factors, the obtained values by xMAP array were comparable to the outline obtained by the ELISA analysis.

**Figure 2:**
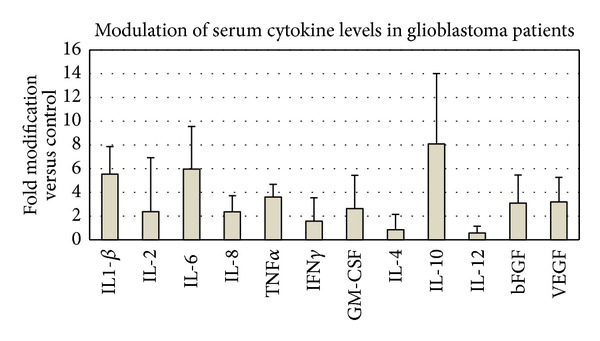
Modulation of serum cytokine levels in glioblastoma patients. The data represent group averages of fold modification versus controls + standard deviations. Statistical significance (one way ANOVA): pro-inflammatory cytokines, *P* < 0.05 for IL-1*β*, IL-6, and TNF*α*, GM-CSF; anti-inflammatory cytokines, *P* < 0.05 for IL-4, and IL-10; angiogenic factors, *P* < 0.05 for bFGF and VEGF. Expression levels of IL-2, IL-8, IFN-*γ*, and IL-12 were modified, but with low statistical significance.

Based on xMAP analysis, the changes in average serum levels (compared to the controls) are presented in Figure 2. 

**Figure 3:**
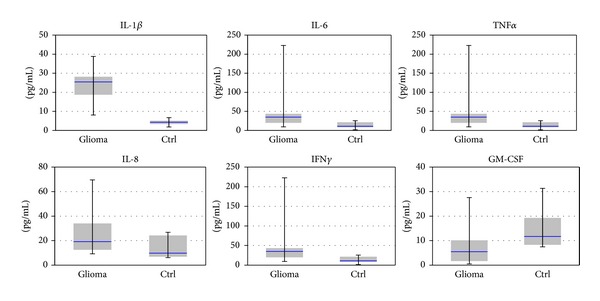
Serum levels of pro-inflammatory cytokines xMAP analysis. Statistical significance (one way Anova): *P* < 0.05 for over-expression of IL-1*β*, TNF*α*, IL-6, and GM-CSF in sera from glioblastoma patients compared to control.

**Figure 4:**
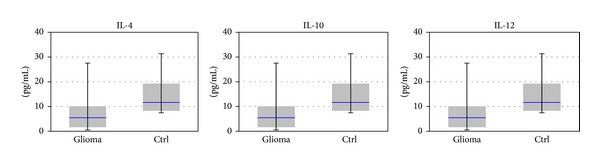
Expression levels of anti-inflammatory cytokines by xMAP analysis. Statistical significance (one way Anova): *P* < 0.05 for IL-4 and IL-10 modified levels in patients' sera versus controls.

**Figure 5:**
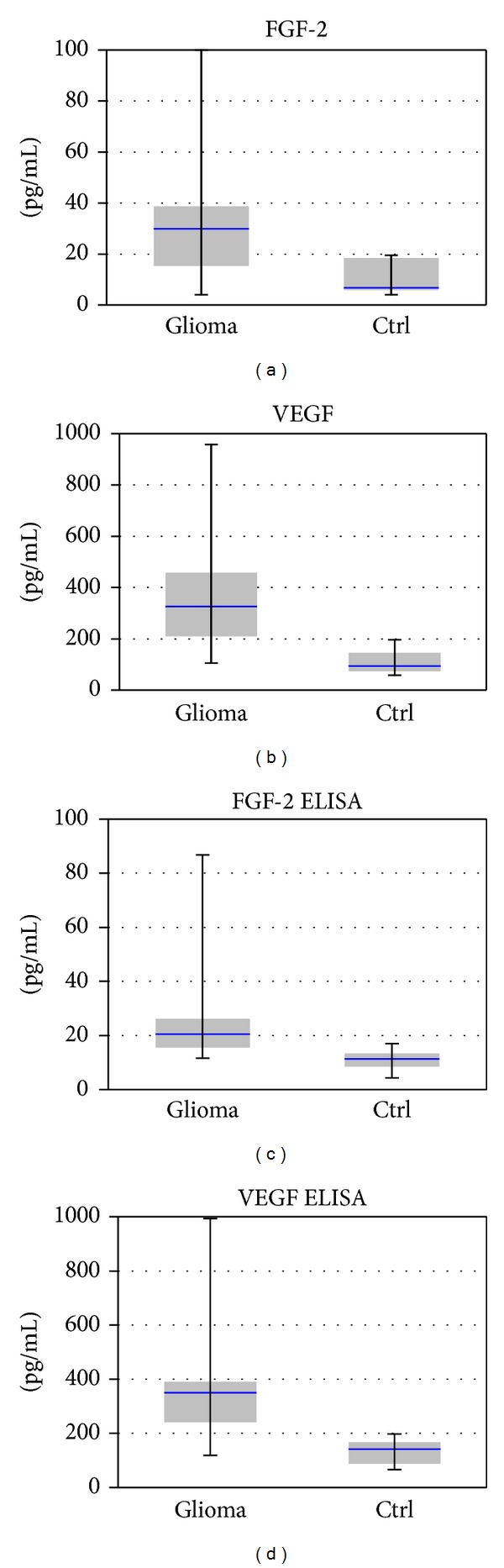
Expression levels of angiogenic factors, by xMAP analysis (a and b) and ELISA (c and d). Statistical significance (one way Anova): *P* < 0.05 for bFGF and VEGF over-expression in sera from glioblastoma patients versus control, by both methods.

Several molecules display a modification in plasma levels of more than 2-fold, which is, as a general practice, a criterion of acceptance as potential marker. However, it also appears evident that, for several cytokines, the intervals of variations in patients were broad. Further details on expression are provided in Figures 3, 4, and 5, where the distributions can be better examined and also cover the behaviour of controls.

 The enhanced expression of IL-1*β* appears to directly correlate with IL-6 and IL-8 levels and inversely correlate with IL-4. In brain, IL-1*β* regulates survival and invasiveness of glioblastoma cells, and anti-IL-1*β* antibodies inhibit both the growth and invasion of glioblastoma cells [17]. Wang et al. showed that in LN-229 glioma cell line, IL-1*β* and TGF-*β* can induce glioma stem cells phenotype and contribute to carcinogenesis [18]. Enhanced secretion of IL-1*β*, IL-6, and IL-8 by glioma cells was reported by Yeung et al. [19], and these cytokines are related with the expansion of GBM (glioblastoma multiforme). In other types of cancers (gastric and oesophageal), IL-1*β* was involved in carcinogenesis and proliferation and played a crucial role in the development of chemical carcinogen-induced tumours [20, 21].

IL-6 appeared overexpressed (average 4-fold) in glioblastoma patients. The determined serum levels are consistent with the ability of tumour cell to secrete pro-inflammatory cytokines, as well as with IL-6 role in stimulation of angiogenesis. According to our data, IL-6 expression correlates with IL-1*β*, IL-8, and IFN-*γ*. Ancrile et al. showed IL-6 is involved in carcinogenesis by angiogenesis and tumour growth and may be a potential anti-invasion target [22]. In U251, T98G and U87 MG glioblastoma cell lines, IL-6 promotes vascular endothelial cell migration and facilitates tumour angiogenesis and invasion [23, 24]. Amplification of the IL-6 gene in patients with glioblastoma multiforme is correlated with decreased survival [25]. 

Serum levels of TNF*α* appeared significantly enhanced (*P* = 2.5*E* − 8), suggesting a strong correlation with the disease; however, the correlation with other molecules is not so strong, suggesting its implication in distinct/complementary regulatory cascades. Hagemann et al. suggested that TNF is involved in tumour cell invasion through upregulation of migration-inhibitory factor (MIF) and through enhanced MMPs production in tumour cells via NF-**κ** *β*- and JNK-signalling [26]. In ovarian cancer, TNF*α* stimulated other cytokines (IL-6), angiogenic factors (VEGF), and chemokines (CCL2 and CXCL12) that promoted tumour growth and metastases [27]. Other studies have showed that TNF over-expression enhances migration and metastasis through induction of CXCR4, MCP-1, and IL-8 and matrix metalloproteinase [28, 29]. Recent studies on U373MG and C6 human glioma cell lines showed that TNF-*α* induces IL-6 synthesis through the JAK/STAT3 pathway and TNF inhibitors can reduce tumour cell invasion [30, 31].

IL-8 has been found to be up-regulated (fold stimulation 1.9) in patient sera, compared to controls. Many studies showed that IL8 is upregulated in gliomas and is involved in the promotion of angiogenesis. In PTEN-deficient glioblastoma cells, repression of IL-8 can inhibit glioblastoma cell proliferation and invasiveness [32]. 

Studies on murine models showed that transplanted glioblastoma tumour cells, which express high levels of IL-2, IL-4, or GM-CSF show enhanced tumour survival. U87-MG glioblastoma cell line expressed high levels of GM-CSF, and GM-CSF over-expression is found exclusively in cultures derived from astrocytomas [33]. 

In glioblastoma patients, IL-2 overexpression averaged 2-fold, but the patient group has been distributed in one subgroup of patients (55%) who displayed strongly enhanced levels of expression (up to 5-fold) while 45% of the patients displayed a moderate enhancement of expression—on average 1.4-fold increase. The distribution could not be yet correlated with other clinical data. Statistical analysis (*t*-test) suggests that the two subgroups represent distinct subpopulation of glioblastoma patients, and so further investigations and integration of more data is required to consolidate and explain this segregation. IL-2 is reported in use for cancer treatment, as a stimulator of T-cell mediated anti-tumour activity. 

Anti-inflammatory cytokines IL-4 and IL-12 appear at lower level in patient's sera at 57–80% compared to controls group (Figure 3). IL-4 is involved in inhibition of cell proliferation, regulation of adhesion molecules, and induction JAK/STAT signalling; IL-4 receptor is overexpressed in malignant glioma cell lines and tumour specimens from patients with glioblastoma, but his mechanism is still unclear [34]. Many studies on murine models showed that IL-12 is a powerful anticancer factor which can inhibit growth of implanted glioblastoma and the increase survival time [35].

IL-10 levels are significantly increased (*P* < 0.001). At a first glance, the strong increase in IL-10 looks paradoxical in the general balance of pro- and anti-inflammatory cytokines; however, the finding confirms previous reports of Kumar et al. [36], who reported significant increase of IL-10 serum levels in patients with anaplastic astrocytoma and glioblastoma. The increase in serum levels of IL-10 may also be correlated with glioma induced immunosuppression. The same study also detected significant decrease in serum levels of glioblastoma and anaplastic astrocytoma, suggesting a systemic impact of brain tumours on the immune system. RT-PCR and immunoassay studies on glioblastoma showed that IL-10 expression is significantly higher in stem-cell-derived tumour sphere cells than in primary cultured glioma cells from the same tumour [37]. IL-10 is significantly overexpressed in high grade tumours and can contribute to progression of astrocytomas [38].

Serum levels of angiogenic factors were considerably elevated in glioblastoma patients, as measured by xMAP analysis and confirmed by ELISA; both VEGF and bFGF were significantly overexpressed (bFGF, 3.05-fold modification, *P* = 0.002, VEGF, 3.2-fold modification, *P* = 0.005), see Figure 5. The expression and distribution profiles of angiogenic factors were similar in both detection methods, with an increase for bFGF of 2.99-fold in xMAP analysis and 3.22 in ELISA and for VEGF of 3.12 and 3.08, respectively.

In GBM, VEGF-VEGFR2 signalling is maintained by continuous secretion of VEGF ligand and promotes tumour growth, invasiveness and enhanced resistance to some treatments [39]. Anti-VEGF therapy and VEGFR inhibitors can delay progression of glioblastoma, but this mechanism is not well understood [40]. *In vitro *and* in vivo *studies showed that stem-cell-like glioma cells secrete elevated levels of VEGF induced by hypoxia, and anti-VEGF therapy cancel proangiogenic effects of glioma [41–43]. 

FGF-2 is involved in neoplastic transformation of glioma cells by activating Ras/Raf/ERK signalling and can stimulate angiogenesis in glioblastoma [44, 45]. 

 Our study was primarily aimed on the estimation of serum level of several cytokines and angiogenic factors in glioblastoma patients, in order to assess their suitability as diagnostic, prognostic and monitoring biomarkers. Nevertheless, many components of the investigated panel are pleiotropic molecules, and, besides their primary role as regulators of cell behaviour, they also play major roles in inflammation and/or tumour related pain. The serum levels of IL-1*β*, TNF*α*, IFN-*γ*, and GM-CSF were significantly increased in glioblastoma patients. A previous study of Makimura et al. [46] investigated the plasma levels of 26 cytokines in cancer patients in correlation with responses to morphine treatment; they could not correlate the levels with pain levels, but were able to correlate some of the investigated molecules with responsiveness to morphine treatment. According to Kawasaki et al. [47] TNF and IL-1*β* cause an increase in the activity of AMPA (2-amino-3-(3-hydroxy-5-methyl-isoxazol-4-yl) propanoic acid) or NMDA (N-methyl-D-aspartate) receptors, while IL-1*β* and IL-6 inhibit gamma-amino-butyric acid (GABA) and glycine-induced ion currents in Rexed lamina II nociceptors, demonstrating that these pro-inflammatory cytokines favour the increase in neuronal excitability. Several studies showed that pro-inflammatory and anti-inflammatory cytokines dysregulation is associated with carcinogenesis and tumour progression of solid cancers, like pancreatic and colorectal [48–50].

## 4. Conclusion

Our findings demonstrate that cytokines and angiogenic factors levels are closely linked to the brain tumour behaviour.

Out of all potential biomarkers for glioblastoma staging and prognostic, a panel of inflammatory cytokines and angiogenic factors is more relevant than single molecules, as proven by our study. Moreover, further investigation could generate a multimolecular panel for better patient stratification and more adequate therapeutical approaches. The involvement of cytokines in inflammation and pain, as well as the relevance of pain in glioblastoma makes them reliable targets for investigation, with potential diagnostics and therapeutic applications. 

xMAP technology might be a suitable tool for evaluation of tumoural development. The advantages of xMAP technology could be less invasive techniques, screening for molecular markers, and validation of putative therapeutic targets.

Further analysis on protein expression and signalling, protein interaction networks, associated with the implementation of a clinical panel for pain scoring, may lead to establish more clearly the connection between mediators of inflammation, signaling pathways and targets for tumoural progression, cancer therapy, and cancer pain therapy. 

Pain is a frequent symptom accused by glioblastoma patients and one of the primary signs conducting to investigation and diagnostics, but, unfortunately, most often the diagnostics appear late or too late; the set of cytokines could add in speed and accuracy, making thus possible earlier diagnostics and onset of therapy.

## References

[1] Westermark B (2012). Glioblastoma—a moving target. *Upsala Journal of Medical Sciences*.

[2] Bleeker FE, Molenaar RJ, Leenstra S (2012). Recent advances in the molecular understanding of glioblastoma. *Journal of Neuro-Oncology*.

[3] Balkwill F, Mantovani A (2001). Inflammation and cancer: back to Virchow?. *The Lancet*.

[4] Colotta F, Allavena P, Sica A, Garlanda C, Mantovani A (2009). Cancer-related inflammation, the seventh hallmark of cancer: links to genetic instability. *Carcinogenesis*.

[5] Larghi CPP, Riboldi E, Allavena P, Mantovani A, Sica A, Baruch AB (2012). Tumor-infiltrating inflammatory cells as possible therapeutic targets. *The Inflammatory Milieu of Tumors: Cytokines and Chemokines That Affect Tumor Growth and Metastasis*.

[6] Sato A, Sakurada K, Kumabe T (2010). Association of stem cell marker CD133 expression with dissemination of glioblastomas. *Neurosurgical Review*.

[7] Pistol-Tanase C, Raducan E, Dima SO (2008). Assessment of soluble angiogenic markers in pancreatic cancer. *Biomarkers in Medicine*.

[8] Iwami K, Natsume A, Wakabayashi T (2011). Cytokine networks in glioma. *Neurosurgical Review*.

[9] de Palma M, Venneri MA, Galli R (2005). Tie2 identifies a hematopoietic lineage of proangiogenic monocytes required for tumor vessel formation and a mesenchymal population of pericyte progenitors. *Cancer Cell*.

[10] Seifert J, Naumann E, Hewig J, Hagemann D, Bartussek D (2006). Motivated executive attention—incentives and the noise-compatibility effect. *Biological Psychology*.

[11] Candi E, Knight RA, Spinedi A, Guerrieri P, Melino G (1997). A possible growth factor role of IL-6 in neuroectodermal tumours. *Journal of Neuro-Oncology*.

[12] Zhu Z, Zhong S, Shen Z (2011). Targeting the inflammatory pathways to enhance chemotherapy of cancer. *Cancer Biology and Therapy*.

[13] Grivennikov SI, Greten FR, Karin M (2010). Immunity, inflammation, and cancer. *Cell*.

[14] Reyes-Gibby CC, Wang J, Spitz M, Wu X, Yennurajalingam S, Shete S (2012). Genetic variations in interleukin-8 and interleukin-10 are associated with pain, depressed mood, and fatigue in lung cancer patients. *Journal of Pain and Symptom Management*.

[15] Wang XM, Wu TX, Hamza M, Ramsay ES, Wahl SM, Dionne RA (2007). Rofecoxib modulates multiple gene expression pathways in a clinical model of acute inflammatory pain. *Pain*.

[16] Milligan E, Zapata V, Schoeniger D (2005). An initial investigation of spinal mechanisms underlying pain enhancement induced by fractalkine, a neuronally released chemokine. *European Journal of Neuroscience*.

[17] Paugh BS, Bryan L, Paugh SW (2009). Interleukin-1 regulates the expression of sphingosine kinase 1 in glioblastoma cells. *The Journal of Biological Chemistry*.

[18] Wang L, Liu Z, Balivada S (2012). Interleukin-1*β* and transforming growth factor-cooperate to induce neurosphere formation and increase tumorigenicity of adherent LN-229 glioma cells. *Stem Cell Research and Therapy*.

[19] Yeung YT, Bryce NS, Adams S (2012). p38 MAPK inhibitors attenuate pro-inflammatory cytokine production and the invasiveness of human U251 glioblastoma cells. *Journal of Neuro-Oncology*.

[20] Tu S, Bhagat G, Cui G (2008). Overexpression of interleukin-1*β* induces gastric inflammation and cancer and mobilizes myeloid-derived suppressor cells in mice. *Cancer Cell*.

[21] Krelin Y, Voronov E, Dotan S (2007). Interleukin-1*β*-driven inflammation promotes the development and invasiveness of chemical carcinogen-induced tumors. *Cancer Research*.

[22] Ancrile B, Lim K, Counter CM (2007). Oncogenic Ras-induced secretion of IL6 is required for tumorigenesis. *Genes and Development*.

[23] Liu Q, Li G, Li R (2010). IL-6 promotion of glioblastoma cell invasion and angiogenesis in U251 and T98G cell lines. *Journal of Neuro-Oncology*.

[24] Li R, Li G, Deng L (2010). IL-6 augments the invasiveness of U87MG human glioblastoma multiforme cells via up-regulation of MMP-2 and fascin-1. *Oncology Reports*.

[25] Tchirkov A, Khalil T, Chautard E (2007). Interleukin-6 gene amplification and shortened survival in glioblastoma patients. *The British Journal of Cancer*.

[26] Hagemann T, Wilson J, Kulbe H (2005). Macrophages induce invasiveness of epithelial cancer cells via NF-*κ*B and JNK. *Journal of Immunology*.

[27] Kulbe H, Thompson R, Wilson JL (2007). The inflammatory cytokine tumor necrosis factor-*α* generates an autocrine tumor-promoting network in epithelial ovarian cancer cells. *Cancer Research*.

[28] Kulbe H, Hagemann T, Szlosarek PW, Balkwill FR, Wilson JL (2005). The inflammatory cytokine tumor necrosis factor-*α* regulates chemokine receptor expression on ovarian cancer cells. *Cancer Research*.

[29] Pollard JW (2004). Tumour-educated macrophages promote tumour progression and metastasis. *Nature Reviews Cancer*.

[30] Ryu J, Ku BM, Lee YK (2011). Resveratrol reduces TNF-*α*-induced U373MG human glioma cell invasion through regulating NF-*κ*B activation and uPA/uPAR expression. *Anticancer Research*.

[31] Tanabe K, Matsushima-Nishiwaki R, Yamaguchi S, Iida H, Dohi S, Kozawa O (2010). Mechanisms of tumor necrosis factor-*α*-induced interleukin-6 synthesis in glioma cells. *Journal of Neuroinflammation*.

[32] de la Iglesia N, Konopka G, Lim K (2008). Deregulation of a STAT3-interleukin 8 signaling pathway promotes human glioblastoma cell proliferation and invasiveness. *Journal of Neuroscience*.

[33] Curran CS, Evans MD, Bertics PJ (2011). GM-CSF production by glioblastoma cells has a functional role in eosinophil survival, activation, and growth factor production for enhanced tumor cell proliferation. *Journal of Immunology*.

[34] Kawakami M, Kawakami K, Puri RK (2003). Interleukin-4-Pseudomonas exotoxin chimeric fusion protein for malignant glioma therapy. *Journal of Neuro-Oncology*.

[35] Chiu TL, Wang MJ, Su CC (2012). The treatment of glioblastoma multiforme through activation of microglia and TRAIL induced by rAAV2-mediated IL-12 in a syngeneic rat model. *Journal of Biomedical Science*.

[36] Kumar R, Kamdar D, Madden L (2006). Th1/Th2 cytokine imbalance in meningioma, anaplastic astrocytoma and glioblastoma multiforme patients. *Oncology Reports*.

[37] Qiu B, Zhang D, Wang C (2011). IL-10 and TGF-*β*2 are overexpressed in tumor spheres cultured from human gliomas. *Molecular Biology Reports*.

[38] Huettner C, Paulus W, Roggendorf W (1994). Increased amounts of IL-10 mRNA in anaplastic astrocytomas and glioblastoma multiforme. *Verhandlungen der Deutschen Gesellschaft fur Pathologie*.

[39] Hamerlik P, Lathia JD, Rasmussen R (2012). Autocrine VEGF-VEGFR2-Neuropilin-1 signaling promotes glioma stem-like cell viability and tumor growth. *Journal of Experimental Medicine*.

[40] Piao Y, Liang J, Holmes L (2012). Glioblastoma resistance to anti-VEGF therapy is associated with myeloid cell infiltration, stem cell accumulation, and a mesenchymal phenotype. *Neuro-Oncology*.

[41] Bao S, Wu Q, Sathornsumetee S (2006). Stem cell-like glioma cells promote tumor angiogenesis through vascular endothelial growth factor. *Cancer Research*.

[42] Chiao MT, Yang YC, Cheng WY, Shen CC, Ko JL (2011). CD133+ glioblastoma stem-like cells induce vascular mimicry in vivo. *Current Neurovascular Research*.

[43] Xu C, Wu X, Zhu J (2013). VEGF promotes proliferation of human glioblastoma multiforme stem-like cells through VEGF receptor 2. *The Scientific World Journal*.

[44] Loilome W, Joshi AD, ap Rhys CMJ (2009). Glioblastoma cell growth is suppressed by disruption of fibroblast growth factor pathway signaling. *Journal of Neuro-Oncology*.

[45] Dunn GP, Rinne ML, Wykosky J (2012). Emerging insights into the molecular and cellular basis of glioblastoma. *Genes and Development*.

[46] Makimura C, Arao T, Matsuoka H (2011). Prospective study evaluating the plasma concentrations of twenty-six cytokines and response to morphine treatment in cancer patients. *Anticancer Research*.

[47] Kawasaki Y, Zhang L, Cheng J, Ji R (2008). Cytokine mechanisms of central sensitization: distinct and overlapping role of interleukin-1*β*, interleukin-6, and tumor necrosis factor-*α* in regulating synaptic and neuronal activity in the superficial spinal cord. *Journal of Neuroscience*.

[48] Momi N, Kaur S, Krishn SR, Batra SK (2012). Discovering the route from inflammation to pancreatic cancer. *Minerva Gastroenterologica e Dietologica*.

[49] Ullman TA, Itzkowitz SH (2011). Intestinal inflammation and cancer. *Gastroenterology*.

[50] Dima SO, Tanase C, Albulescu R (2012). An exploratory study of inflammatory cytokines as prognostic biomarkers in patients with ductal pancreatic adenocarcinoma. *Pancreas*.

